# Mitochondrial Dynamics in Aging Heart

**DOI:** 10.3390/biomedicines13112603

**Published:** 2025-10-24

**Authors:** Pankaj Patyal, Gohar Azhar, Ambika Verma, Shakshi Sharma, Jyotsna Shrivastava, Sayed Aliul Hasan Abdi, Xiaomin Zhang, Jeanne Y. Wei

**Affiliations:** Donald W. Reynolds Department of Geriatrics and Institute on Aging, University of Arkansas for Medical Sciences, Little Rock, AR 72205, USA; ppatyal@uams.edu (P.P.); azhargohar@uams.edu (G.A.); averma@uams.edu (A.V.); ssharma@uams.edu (S.S.); jshrivastava@uams.edu (J.S.); shasan@uams.edu (S.A.H.A.); zhangxiaomin@uams.edu (X.Z.)

**Keywords:** mitochondria, cardiac aging, apoptosis, sirtuins, mtDNA, interventions

## Abstract

Aging is a major risk factor for cardiovascular disease, driving progressive structural and functional decline of the myocardium. Mitochondria, the primary source of ATP through oxidative phosphorylation, are essential for cardiac contractility, calcium homeostasis, and redox balance. In the aging heart, mitochondria show morphological alterations including cristae disorganization, swelling, and fragmentation, along with reduced OXPHOS efficiency. These defects increase proton leak, lower ATP production, and elevate reactive oxygen species (ROS), causing oxidative damage. Concurrent disruptions in mitochondrial fusion and fission further impair turnover and quality control, exacerbating mitochondrial dysfunction and cardiac decline. Serum response factor (SRF) signaling, a crucial regulator of cytoskeletal and metabolic gene expression, plays a key role in modulating mitochondrial function during cardiac aging. Dysregulation of SRF impairs mitochondrial adaptability, contributing to dysfunction. Additionally, reduced levels of nicotinamide adenine dinucleotide (NAD^+^) hinder sirtuin-dependent deacetylation, further compromising mitochondrial efficiency and stress resilience. These cumulative defects activate regulated cell death pathways, leading to cardiomyocyte loss, fibrosis, and impaired diastolic function. Mitochondrial dysfunction therefore serves as both a driver and amplifier of cardiac aging, accelerating the transition toward heart failure. This narrative review aims to provide a comprehensive overview of mitochondrial remodeling in the aging myocardium, examining the mechanistic links between mitochondrial dysfunction and myocardial injury. We also discuss emerging therapeutic strategies targeting mitochondrial bioenergetics and quality control as promising approaches to preserve cardiac function and extend cardiovascular health span in the aging population.

## 1. Introduction

Cardiac aging is a complex, multifactorial process characterized by progressive structural and functional decline of the myocardium, culminating in increased susceptibility to heart failure and other cardiovascular diseases [1,2]. Among the cellular organelles implicated in this decline, mitochondria play a pivotal role due to their central function in adenosine triphosphate (ATP) generation, calcium handling, and redox regulation [3,4]. The adult heart derives the vast majority of its ATP from mitochondrial oxidative phosphorylation (OXPHOS), a process that is highly dependent on the integrity of the electron transport chain (ETC) and the structural organization of mitochondrial cristae [5]. With advancing age, cardiac mitochondria undergo profound morphological and functional alterations, including cristae disruption, swelling, fragmentation, and vacuolization, accompanied by a decline in ETC activity, particularly complexes I and IV, leading to impaired OXPHOS efficiency and bioenergetic insufficiency [6]. These alterations are closely intertwined with disruptions in mitochondrial dynamics, where the balance between fusion and fission is lost, favoring excessive fragmentation and reduced network connectivity [7]. Defective mitochondrial quality control, encompassing impaired mitophagy and diminished biogenesis, further exacerbates the accumulation of dysfunctional organelles [8]. The resulting energetic failure is compounded by increased reactive oxygen species (ROS) production, oxidative damage to mitochondrial DNA (mtDNA), and maladaptive activation of inflammatory signaling pathways, creating a self-perpetuating cycle of mitochondrial decline and cellular injury [9,10]. In aged cardiomyocytes, mitochondrial dysfunction not only compromises contractile performance but also orchestrates multiple regulated cell death pathways, including apoptosis, necroptosis, pyroptosis, and ferroptosis, which contribute to cumulative cardiomyocyte loss and pathological remodeling [11,12]. Emerging evidence highlights the critical involvement of mitochondrial sirtuins, amino acid metabolism, and transcriptional regulation in maintaining mitochondrial homeostasis during aging, offering promising therapeutic entry points [13,14,15]. Strategies aimed at restoring mitochondrial integrity, through enhancing mitophagy, promoting biogenesis, modulating dynamics, and targeting redox balance, are increasingly recognized as viable approaches to mitigate age-related cardiac dysfunction. Small molecules, caloric restriction, sirtuin activators, and novel gene therapy strategies are being explored to delay or reverse mitochondrial and cardiac aging [16,17,18].

In this review, we first describe the morphological and functional alterations in mitochondria observed in the aging heart, with emphasis on OXPHOS efficiency, cristae integrity, and bioenergetic decline. We then examine changes in mitochondrial dynamics and quality control mechanisms, highlighting their contribution to the accumulation of dysfunctional organelles. The review further explores multiple regulated cell death mechanisms, involvement of sirtuins and metabolic adaptations, particularly in amino acid metabolism, and their influence on mitochondrial health. Finally, we discuss the emerging therapeutic strategies aimed at mitigating age-associated mitochondrial deterioration to preserve cardiac function and extend health span.

## 2. Mitochondrial Morphological and Functional Alterations in the Aging Heart

Mitochondria are central to cardiac physiology, as the heart demands a continuous and substantial supply of ATP to sustain contractile function [19]. These organelles are the primary sites of OXPHOS, a highly efficient process coupling electron transport to ATP synthesis [20,21]. However, with advancing age, mitochondria in cardiac tissue undergo profound morphological and functional changes that compromise their bioenergetic capacity [22,23]. These alterations contribute to diminished cardiac performance and increased vulnerability to age-related cardiovascular diseases. Mitochondrial morphology in the aging heart is characterized by several distinct ultrastructural abnormalities [24]. Electron microscopy studies consistently reveal that aged cardiac mitochondria exhibit swelling, disrupted or less dense cristae, fragmentation, and vacuolization [25]. The inner mitochondrial membrane cristae contain the protein complexes of the electron transport chain (ETC) and ATP synthase, which are essential for efficient oxidative phosphorylation (OXPHOS). Disruption of these cristae markedly impairs mitochondrial function, as a reduction in cristae density decreases the surface area available for OXPHOS, thereby lowering ATP production efficiency [26]. While earlier TEM studies of cardiac tissue from aged rats reported no significant age-associated alterations in mitochondrial cristae structure [27], more recent clinical and experimental investigations have shown that aging hearts often exhibit swollen mitochondria with disrupted or fragmented inner membrane cristae [28]. Additionally, aged mitochondria often show impaired interactions with other cellular organelles such as the endoplasmic reticulum, which can affect calcium handling and energy metabolism [29]. Taken together, these morphological changes highlight the vulnerability of mitochondrial structure in aging cardiac tissue and the downstream effects on cellular bioenergetics. Aging is associated with a decline in the efficiency and capacity of OXPHOS in cardiac mitochondria, directly affecting myocardial energy homeostasis [30]. Interestingly, some studies have reported that aging has little or no effect on cardiac mitochondrial function [31], with such discrepancies largely attributed to variations in the experimental techniques used to assess mitochondrial performance. However, more recent advances in analytical methods and experimental models generally support the view that mitochondrial function does, in fact, decline with cardiac aging. Multiple studies demonstrate that the activity of key ETC complexes, particularly complex I (NADH: ubiquinone oxidoreductase) and complex IV (cytochrome c oxidase), is significantly reduced in aged cardiac mitochondria [32]. Interestingly, aging-associated defects in mitochondrial ATP synthesis are not uniform across all mitochondria in the heart. Subpopulations such as subsarcolemmal mitochondria (located beneath the plasma membrane) and interfibrillar mitochondria (interspersed among myofibrils) may display differential susceptibility to age-related damage, influencing overall cardiac bioenergetics heterogeneity [33]. Morphological integrity and functional capacity of mitochondria are intimately connected. Disruptions in cristae structure and membrane integrity directly impair ETC complex assembly and function, reducing OXPHOS efficiency. The combination of morphological disarray and functional impairment therefore creates a vulnerable mitochondrial pool within aged cardiomyocytes [34,35]. These dysfunctional mitochondria fail to meet energy demands, generate damaging ROS, and contribute to myocardial aging and pathology.

## 3. Mitochondrial Turnover and Quality Control in Cardiac Aging

**Figure 1:**
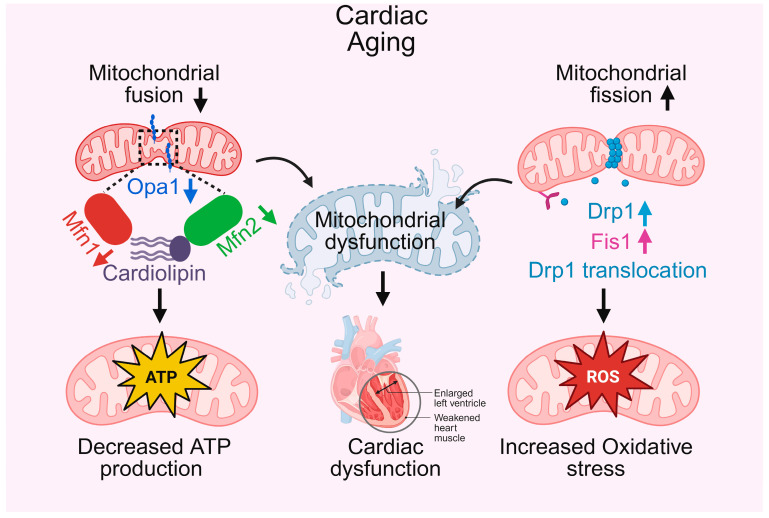
Mitochondrial fusion and fission in cardiac aging. This schematic illustrates how alterations in mitochondrial fusion and fission contribute to cardiac aging. On the left, decreased mitochondrial fusion, mediated by downregulation of Opa1, Mfn1, and Mfn2, disrupts cardiolipin integrity, leading to decreased ATP production and impaired cardiac energy supply. On the right, altered mitochondrial fission, characterized by upregulation of Drp1, Fis1, and Drp1 translocation, increases reactive oxygen species (ROS) generation, contributing to oxidative stress. These changes collectively result in mitochondrial dysfunction, which underlies cardiac dysfunction, manifested as enlarged left ventricle and weakened heart muscle.

In the aging heart, the intricate balance of mitochondrial dynamics, comprising fusion and fission, is progressively lost, leading to profound structural and functional deficits in heart [36]. Mitochondrial fusion, which promotes network connectivity, content mixing, and metabolic efficiency, is impaired with age due to decreased expression of key mediators such as Mitofusin 2 (Mfn2) and Optic Atrophy 1 (Opa1) [37,38], as well as alterations in cardiolipin, a lipid critical for maintaining inner mitochondrial membrane integrity [39]. These changes hinder mitochondrial elongation and cristae remodeling, thereby compromising OXPHOS and contributing to reduced ATP production, as depicted in Figure 1. In parallel, mitochondrial fission becomes excessively activated through enhanced Dynamin-related protein 1 (Drp1) translocation and upregulation of Fission 1 (Fis1), resulting in a fragmented mitochondrial network prone to depolarization and ROS overproduction [40,41]. This shift toward fission-dominant morphology not only disrupts mitochondrial ultrastructure but also increases oxidative stress, further damaging mitochondrial and cellular components. Furthermore, mitochondrial quality control pathways especially PINK1/Parkin-mediated mitophagy become dysfunctional [42]. While aged cardiomyocytes may transiently upregulate PINK1 and Parkin under stress, the overall mitophagic flux is blunted, leading to inefficient clearance of damaged mitochondria [43]. Mitochondrial biogenesis is also impaired due to the downregulation of transcriptional coactivators such as PGC-1α and NRF1, resulting in a net decline in mitochondrial number and respiratory capacity [44]. Disruption of ER–mitochondrial contacts in aged cells further impairs calcium handling and intracellular communication, exacerbating contractile dysfunction and instability [45]. Together, these age-associated alterations, fragmented mitochondria, impaired mitophagy, reduced biogenesis, energy failure, and increased ROS generation converge to drive mitochondrial dysfunction, a central hallmark of cardiac aging.

## 4. Mitochondrial ROS and mtDNA

Mitochondria are the primary generators of ROS especially from electron leakage at complex III’s Qo site, which can precede and drive age-related cardiac dysfunction [46]. In aging hearts, diminished electron transport chain integrity and reduced assembly of respirasomes further elevate ROS production [47]. Additional sources of mitochondrial ROS include p66Shc and monoamine oxidase (MAO); notably, age-associated increases in MAO-B contribute markedly to oxidative damage in aged cardiac tissue [48]. Importantly, mtROS not only impair energy metabolism but also trigger inflammatory signaling, including activation of NF κB, which drives proinflammatory cytokine expression in aging cardiac cells [49,50]. Mitochondrial DNA (mtDNA) is especially vulnerable to oxidative damage due to its proximity to ROS-generating sites and lack of protective histones [51]. Oxidative lesions and mutations in mtDNA accumulate over time, contributing to compromised oxidative phosphorylation and disrupted mitochondrial dynamics, thereby reinforcing a harmful feedback loop of ROS overproduction and mitochondrial dysfunction. Moreover, mtDNA fragments escaping into the cytosol or circulation can act as damage-associated molecular patterns (DAMPs), activating the cGAS–STING and NLRP3 inflammasome pathways, promoting chronic inflammation, an emerging hallmark of age-related cardiovascular dysfunction [9]. Studies have shown that aging accelerates mtDNA mutation rates, which can impede mitochondrial degradation processes like autophagy, thereby exacerbating mitochondrial dysfunction and promoting inflammation through pathways such as mTOR [52]. Furthermore, the accumulation of somatic mtDNA mutations, often termed cryptic mutations, has been observed in various tissues during aging [53]. These mutations can disrupt mitochondrial homeostasis and contribute to the progression of age-related diseases, including cardiovascular conditions.

## 5. Transcriptional Regulation of Mitochondria in Cardiac Aging

**Figure 2:**
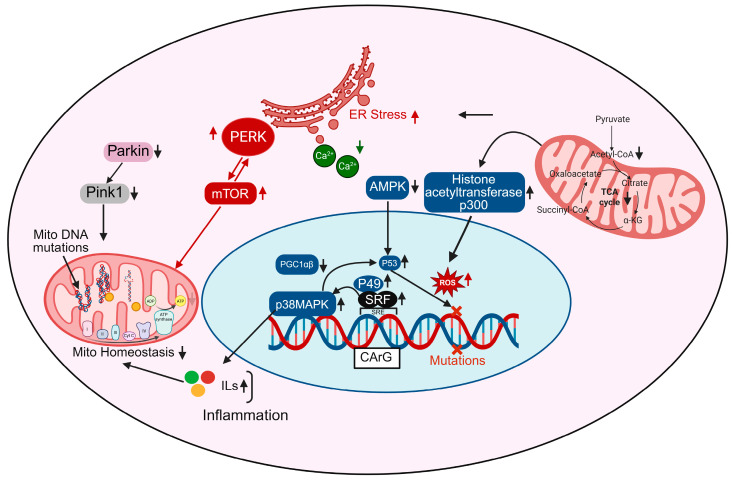
Transcriptional regulation of cellular stress responses: Role of SRF, PGC-1α, and related pathways. Transcription factors such as SRF and P49 regulate gene expression in response to cellular stress and mutations. Their interaction with PGC-1α/β coordinates the activity of key regulators including Parkin, Pink1, and mTOR, which control mitochondrial function and stress responses. PERK signaling and changes in calcium (Ca^2+^) levels contribute to ER stress, while AMPK, p38MAPK, and p53 pathways are involved in metabolic regulation and inflammation. Mutations in mitochondrial DNA, accumulation of reactive oxygen species (ROS), and disruptions in the TCA cycle can impair normal cellular functions.

In aging cardiomyocytes, transcriptional control of mitochondrial homeostasis is profoundly disrupted, contributing to energetic failure, oxidative stress, and maladaptive cardiac remodeling. Central to this regulation are transcription factors and coactivators such as PGC-1α/β (Peroxisome proliferator-activated receptor gamma coactivator 1-alpha/beta), p53, and serum response factor (SRF), which orchestrate mitochondrial biogenesis, antioxidant defenses, and oxidative metabolism [54,55]. Figure 2 depicts the activation of AMP-activated protein kinase (AMPK), a key energy sensor that, when stimulated, promotes mitochondrial biogenesis by upregulating PGC-1β and downstream transcriptional targets involved in oxidative phosphorylation and fatty acid metabolism. However, with aging, AMPK signaling is often suppressed due to increased metabolic stress and inflammation, leading to reduced mitochondrial renewal [56]. p38 MAPK, a stress-activated kinase, is upregulated in aging and activates and also enhances inflammatory gene expression and contributes to mtDNA damage when chronically stimulated [57]. The transcription factor SRF, shown binding to CArG boxes in gene promoters, becomes dysregulated during aging and may interact with co-factors such as p49 and p53, further altering the expression of mitochondrial and cytoskeletal genes [58,59]. In cardiac aging, aberrant SRF signaling can downregulate fusion proteins like MFN2 and OPA1 while upregulating fission proteins such as DRP1, promoting mitochondrial fragmentation, a hallmark of aging cardiomyocytes. SRF also influences the transcription of genes involved in mitochondrial biogenesis, potentially impairing the renewal of healthy mitochondria in the aged heart. Emerging evidence suggests that SRF dysregulation contributes to increased mitochondrial ROS production, loss of membrane potential, and reduced ATP generation, linking transcriptional control directly to mitochondrial dysfunction [55]. By integrating cytoskeletal remodeling with mitochondrial regulation, SRF represents a critical master regulator through which aging-related transcriptional changes translate into bioenergetic decline and cardiomyocyte vulnerability.

ROS accumulation, prominently shown in Figure 2, is both a cause and consequence of transcriptional dysregulation; ROS activate histone acetyltransferases like p300, leading to chromatin remodeling and aberrant gene expression, while also damaging mtDNA directly, thereby compounding mitochondrial dysfunction [60]. Moreover, mtDNA mutations accumulate due to increased ROS and impaired mitophagy (as shown via downregulated Pink1 and Parkin pathways), further compromising the ETC and exacerbating ROS production, a vicious cycle that reinforces transcriptional and metabolic decline [16,61]. mTOR and PERK pathways, elevated in aged cardiomyocytes, contribute to ER stress and impair mitochondrial turnover and energy sensing, further disrupting transcriptional programs that support mitochondrial integrity [62]. Additionally, metabolic intermediates from the TCA cycle like citrate, α-ketoglutarate (α-KG), and acetyl-CoA feed into nuclear epigenetic regulation by modulating histone acetylation and DNA methylation, linking mitochondrial metabolism directly to transcriptional outcomes [63]. In aged hearts, TCA cycle flux is reduced, decreasing the availability of these key metabolites and impairing mitochondrial-nuclear communication [64]. Finally, increased inflammatory signaling feeds back into the transcriptional network, promoting pathological gene expression, nuclear ROS production, and further mitochondrial instability [65]. Altogether, these interconnected transcriptional disruptions lead to impaired mitochondrial biogenesis, increased mtDNA damage, chronic inflammation, and bioenergetic failure, contributing to the structural and functional deterioration of the aging heart.

## 6. Mitochondrial Dysfunction Orchestrates Multimodal Cell Death in Cardiac Aging

**Figure 3:**
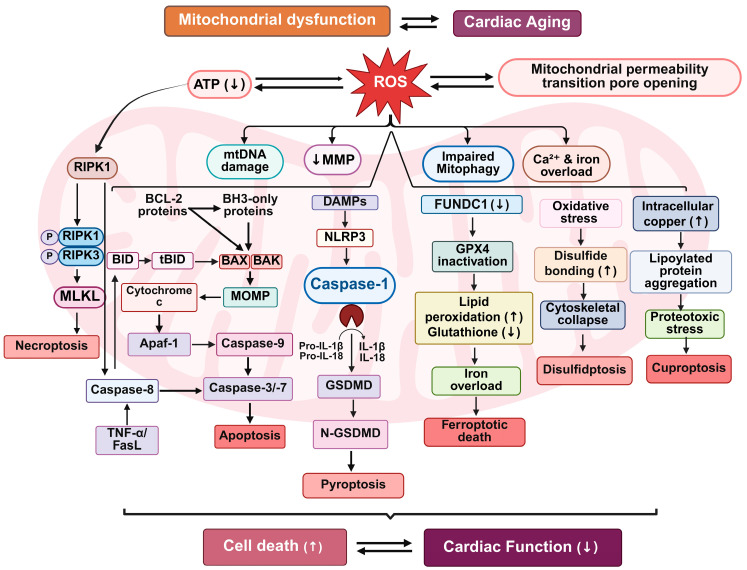
Mitochondrial dysfunction-driven cell death pathways in cardiac aging. This schematic illustrates the interconnected mitochondrial cell death pathways contributing to cardiac aging. Mitochondrial dysfunction and increased ROS generation initiate a cascade of events including reduced mitochondrial membrane potential (↓MMP), impaired mitophagy, mitochondrial permeability transition pore (mPTP) opening, and altered ion homeostasis. These stressors activate multiple regulated cell death pathways: apoptosis (via BCL-2 family proteins and cytochrome c release), necroptosis (via RIPK1/RIPK3/MLKL signaling), pyroptosis (via inflammasome activation and GSDMD cleavage), ferroptosis (via GPX4 inactivation and lipid peroxidation), disulfidptosis (via oxidative protein cross-linking), and cuproptosis (via copper-induced proteotoxicity). These mechanisms collectively lead to increased cell death and declining cardiac function during aging.

Mitochondria play critical roles not only in ATP generation but also in the regulation of programmed cell death pathways in the cardiac aging [66,67]. In the aging myocardium, the deterioration of mitochondrial function integrates bioenergetic failure, oxidative stress, and defective quality control mechanisms, ultimately promoting multimodal cell death and cardiomyocyte loss [5]. Defective mitochondrial quality control mechanisms, including impaired mitophagy and diminished activity of key proteostasis pathways, contribute to the persistence of dysfunctional mitochondria within cardiomyocytes. These damaged organelles can release pro-apoptotic factors such as cytochrome c and activate downstream caspase-dependent and -independent cell death cascades [68]. These mitochondrial perturbations initiate a cascade of interconnected programmed cell death (PCD) mechanisms including apoptosis, ferroptosis, necroptosis, pyroptosis, and recently characterized modalities such as cuproptosis and disulfidptosis that synergistically accelerate cardiac degeneration (Figure 3).

### 6.1. Apoptosis and Mitochondrial Dysfunction

Intrinsic (mitochondrial) apoptosis is a well-established form of programmed cell death activated by internal stress signals such as oxidative damage, DNA damage, and mitochondrial membrane depolarization [69]. Mitochondrial outer membrane permeabilization, regulated by B-cell lymphoma 2 (Bcl-2) family proteins, initiates the release of cytochrome c, second Mitochondria-derived Activator of Caspases (Smac)/DIABLO, and apoptosis-inducing factor (AIF) into the cytosol [70]. Cytochrome c forms the apoptosome complex with Apoptotic protease activating factor 1 (Apaf-1) and procaspase-9, culminating in the activation of effector caspases-3 and -7 [71]. With aging, a shift in the balance between pro- and anti-apoptotic Bcl-2 proteins sensitizes cardiomyocytes to apoptosis [72]. Upregulation of Bcl-2 Homology 3 (BH3) only proteins (e.g., BID, NOXA, PUMA) and downregulation of anti-apoptotic members (Bcl-2, Bcl-xL) promote mitochondrial outer membrane permeabilization [73,74]. Previous studies have demonstrated age-associated differences in the regulation of apoptotic pathways in the heart, including altered expression of Bcl-2 family proteins after ischemic injury [75,76] and highlighted the role of mitochondrial proteins such as BCL-xL in promoting successful aging across species [77,78]. Additionally, the extrinsic apoptotic pathway, mediated by death receptor ligands such as Fas ligand (FasL) and Tumor necrosis factor-α (TNF-α), becomes increasingly active in aged hearts due to chronic inflammation and senescence-associated secretory phenotype [79]. This results in caspase-8 activation and cleavage of BH3 interacting-domain death agonist (BID) to truncated Bid (tBID), reinforcing mitochondrial involvement in extrinsic apoptotic signaling [80].

### 6.2. Necroptosis and Mitochondrial Permeability

When apoptotic pathways are inhibited or exhausted, necroptosis serves as an alternative route to PCD. This regulated necrotic mechanism is orchestrated by receptor-interacting protein kinases RIPK1 and RIPK3, which phosphorylate mixed lineage kinase domain-like pseudo kinase (MLKL) [81]. Activated MLKL disrupts plasma membrane integrity, resulting in lytic cell death [82]. In aging hearts, chronic TNF-α signaling and mitochondrial dysfunction potentiate necroptotic signaling [83]. ROS and Ca^2+^ overload facilitate opening of the mitochondrial permeability transition pore (mPTP), causing ATP depletion and mitochondrial swelling, further amplifying necrotic responses [84].

### 6.3. Pyroptosis and Mitochondrial Inflammasomes

Mitochondrial dysfunction also contributes to inflammasome activation [85,86]. Accumulation of mtDNA and ROS activates the NOD-like receptor family pyrin domain containing 3 (NLRP3) inflammasome, leading to caspase-1–dependent cleavage of gasdermin D (GSDMD) and release of IL-1β and IL-18 [87,88]. GSDMD forms pores in both plasma and mitochondrial membranes, propagating pyroptotic cell death and amplifying inflammation [89,90]. This process links innate immunity and mitochondrial stress in the context of inflammaging.

### 6.4. Mitophagy Impairment and Ferroptosis

Mitophagy, a specialized form of autophagy responsible for removing dysfunctional mitochondria, is compromised in cardiac aging. Reduced expression and activity of mitophagy regulators such as FUN14 domain containing 1 (FUNDC1) impair mitochondrial turnover, exacerbating oxidative damage and metabolic stress [91,92]. Ferroptosis is a distinct, non-apoptotic form of regulated cell death that is iron-dependent and marked by the excessive accumulation of lipid peroxides and the depletion of the antioxidant glutathione. A central regulator of this process is glutathione peroxidase 4 (GPX4), which utilizes glutathione to detoxify phospholipid hydroperoxides, thereby preventing lipid peroxidation and maintaining membrane integrity [93]. Loss of GPX4 function, whether due to decreased expression or impaired mitochondrial import, leads to the unchecked propagation of lipid peroxides, culminating in ferroptotic cell death. In the context of aging cardiomyocytes, mitochondrial reactive oxygen species (ROS), dysregulated iron metabolism, and aberrant lipid processing converge to heighten susceptibility to ferroptosis [94]. These age-associated metabolic and redox imbalances create a cellular milieu conducive to ferroptotic signaling [95].

### 6.5. Cuproptosis and Disulfidptosis

Recent studies have expanded the spectrum of mitochondria-regulated cell death modalities. Cuproptosis, triggered by intracellular copper accumulation, involves aggregation of lipoylated mitochondrial proteins and proteotoxic stress, particularly in the context of disrupted mitochondrial respiration [96]. Similarly, disulfidptosis is driven by excessive disulfide bond formation under oxidative and metabolic stress, causing cytoskeletal collapse and cell death [97]. Both mechanisms have been implicated in age-related degenerative conditions, including cardiac decline.

## 7. Mitochondrial Sirtuins in Cardiac Aging

**Figure 4:**
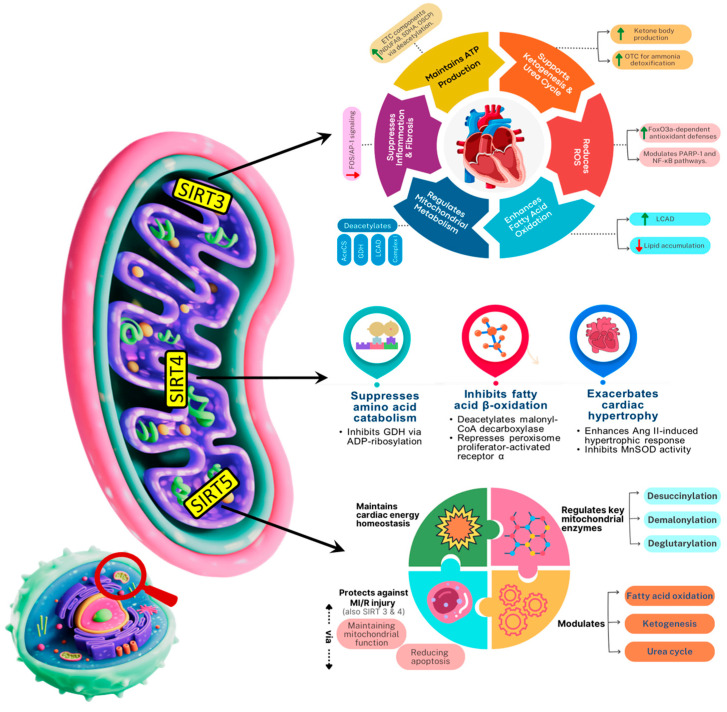
Schematic depicting the roles of Mitochondrial sirtuins (SIRT3, SIRT4, SIRT5) in regulating mitochondrial metabolism, oxidative stress, and aging-related cardiac function. SDHA: Succinate dehydrogenase; OSCP: Oligomycin-Sensitivity Conferring Protein; OTC: Ornithine Transcarbamylase; AceCS: Acetyl-CoA Synthetase; ETC: Electron Transport Chain; GDH: Glutamate Dehydrogenase; PARP1: poly (ADP-ribose) polymerase-1; LCAD: Long-chain Acyl coenzyme A dehydrogenase.

Mitochondrial sirtuins are key modulators of regulated cell death, helping preserve cardiac viability. Sirtuins are the key modulators of regulated cell death by controlling mitochondrial function and metabolism, influencing pathways like apoptosis and ferroptosis. Sirtuins, a family of NAD^+^-dependent deacetylases, play a pivotal role in maintaining mitochondrial health during cardiac aging by regulating mitochondrial biogenesis, dynamics, and stress responses. Of the seven mammalian Sirtuins (SIRT1–7), SIRT3, SIRT4, and SIRT5 are localized to mitochondria and are critical mediators of mitochondrial integrity and functionality as well as cardiac longevity [98,99,100]. SIRT3 is mostly located in mitochondria, with a little presence in the nucleus [101]. The majority of SIRT4 is located in the mitochondrial matrix, with a little amount present in the cytosol and nucleus [102]. SIRT5, like SIRT3 and SIRT4, is mostly found in the mitochondrial matrix, but also exists in the cytosol and nucleus in small amounts [103] and have various functions as summarized in Figure 4. SIRT3 is currently the most studied mitochondrial sirtuin. It plays a pivotal role in mitochondrial functions and aging has been shown to exert cardioprotective effects through multiple mechanisms [104,105]. These include by maintaining basal ATP levels by activating several ETC factors through deacetylation, such as NDUFA9 in complex I, succinate dehydrogenase in complex II, and oligomycin-sensitivity conferring protein in complex V [106,107]. SIRT3 reduces ROS levels in myocardial tissues (thus mitigating cardiac hypertrophy) via augmenting (deacetylating) FoxO3a-dependent antioxidant defense mechanisms [108,109,110]. SIRT3 lowers the level of acetylation and activity of poly (ADP-ribose) polymerase-1 [111] and exert protective effects through NF-κB signaling pathway [112]. Existing evidence also indicates that SIRT3 overexpression partially inhibits the inflammatory and profibrotic impacts in cardiomyocytes by modulating the FOS/activator protein-1 signaling pathway [106,113]. In SIRT3-deficient mice, impaired fatty acid oxidation contributes to reduced ATP levels and heart lipid accumulation. This is linked to loss of deacetylation in multiple mitochondrial metabolic proteins, including acetyl-CoA synthetase 2, glutamate dehydrogenase, long-chain acyl-CoA dehydrogenase (LCAD), and components of the electron transport chain I [114]. Furthermore, SIRT3 promotes ketone body production during fasting thereby reducing metabolic stress and preserving cardiac function [115]. SIRT4 acts in the opposite direction to SIRT3, suppresses amino acid catabolism, GDH inhibition via ADP-ribosylation [116]. It inhibits fatty acid β-oxidation via deacetylating malonyl-coadecarboxylase and by repressing peroxisome-activated receptor a [117]. SIRT5, a mitochondrial NAD^+^-dependent sirtuin, plays a pivotal role in modulating metabolic enzymes through selective post-translational deacylation activities namely desuccinylation, demalonylation, and deglutarylation [118]. These modifications effectively tweak enzyme function and thereby orchestrate key metabolic pathways, including fatty acid β-oxidation, ketogenesis, and the urea cycle, all of which are critical for cardiac energy homeostasis. Furthermore, SIRT5 activates the key urea cycle enzyme CPS1 through deacetylation, enhancing ammonia detoxification, whereas SIRT5 deficiency compromises CPS1 activity and leads to hyperammonemia under stress conditions [119,120]. Given the heart’s reliance on fatty acids and ketone bodies for energy, and the necessity to manage nitrogenous waste, SIRT5′s modulation of these pathways is essential for cardiac energy homeostasis, enabling both efficient energy production and metabolic resilience. Overall, mitochondrial sirtuins (SIRT3, SIRT4, and SIRT5) play pivotal roles in maintaining cardiac function during aging by regulating metabolism, oxidative stress, and mitochondrial quality.

## 8. Therapeutic Potential of Targeting Mitochondria to Combat Age-Related Cardiac Dysfunction

Mitochondria are central to cellular energy production, redox balance, and apoptosis regulation. As organisms age, mitochondrial dysfunction becomes a hallmark, contributing to increased reactive oxygen species production, mtDNA mutations, impaired mitophagy, and altered mitochondrial dynamics. These changes are particularly impactful in cardiac tissue, where mitochondrial health is crucial for maintaining contractile function and metabolic homeostasis [36]. Therefore, addressing mitochondrial dysfunction represents a promising avenue for therapeutic intervention in age-related cardiac dysfunction. These different therapeutic strategies targeting mitochondrial function hold promise for mitigating age-related cardiac dysfunction.

### 8.1. Amino Acid Metabolism in Maintaining Cardiac Mitochondrial Function

Amino acids are essential for maintaining cardiac mitochondrial health, providing the building blocks and energy needed for optimal mitochondrial function. Aging-related declines in amino acid availability and utilization can impair mitochondrial efficiency, contributing to reduced cardiac performance. Supplementation with essential amino acids or high-quality protein has been shown to support mitochondrial function, enhance energy metabolism, and maintain overall cardiac and cellular health [121,122,123]. Branched-chain amino acids (BCAAs), namely leucine, isoleucine, and valine are particularly important in promoting mitochondrial biogenesis and enhancing the functional capacity of cardiac muscle [124,125,126]. These essential amino acids are catabolized within mitochondria by the branched-chain α-keto acid dehydrogenase complex. The resulting metabolites serve as key intermediates in the tricarboxylic acid cycle, directly contributing to ATP production and mitochondrial energy metabolism [127,128].

**Figure 5:**
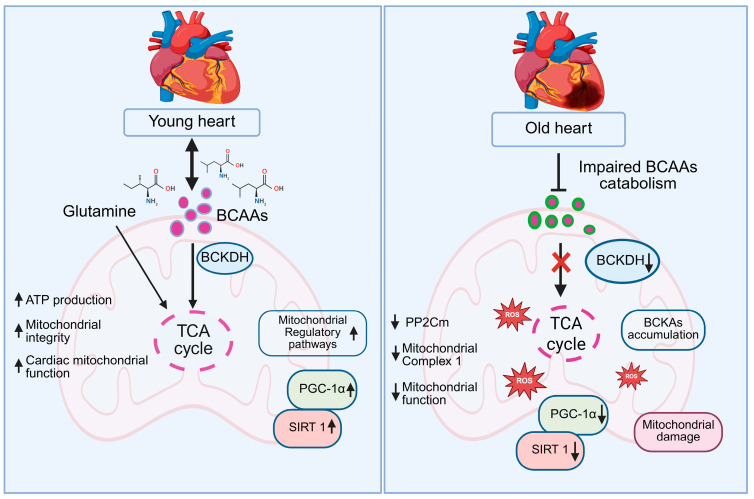
Schematic representation of BCAA metabolism and mitochondrial function in young versus old hearts. In young hearts, efficient BCAA catabolism via BCKDH fuels the TCA cycle, boosting ATP production, mitochondrial integrity, and cardiac function, supported by glutamine, PGC-1α, and SIRT1. However, in old hearts, reduced BCKDH activity impairs BCAA catabolism, causing BCKA accumulation, TCA cycle disruption, ROS production, and mitochondrial damage, with decreased PGC-1α and SIRT1.

Evidence from cell-based experiments, animal models, and clinical studies demonstrates that BCAA supplementation enhances mitochondrial content, improves oxidative phosphorylation efficiency, and supports cardiac contractile performance under both physiological and pathological conditions [129,130]. Further, BCAA-enriched diets also improved myocardial energy metabolism and enzymatic activity in rats subjected to physical stress, underscoring the importance of BCAAs in maintaining cardiac mitochondrial function [131]. BCAAs have been shown to mitigate mitochondrial dysfunction and suppress apoptosis in models of cardiac hypertrophy and diabetic cardiomyopathy, primarily through activation of mitochondrial regulatory pathways involving PGC-1α and SIRT1 [132]. A randomized controlled trial by highlighted the clinical relevance of these findings, showing that BCAA supplementation improved exercise capacity and mitochondrial respiratory function in patients with heart failure [133]. In pathological conditions such as heart failure and cardiomyopathy, impaired BCAA catabolism, often due to downregulation of Protein phosphatase 2Cm (PP2Cm) or reduced BCKDH activity, can lead to the accumulation of branched-chain keto acids (BCKAs), which inhibit mitochondrial complex I, promote excessive reactive oxygen species (ROS) production, and compromise mitochondrial function. PP2Cm plays a crucial role in activating BCKDH by dephosphorylating its inhibitory sites, and its reduced expression in aging disrupts this activation, leading to diminished BCAA oxidation. Furthermore, aging is associated with a decline in BCKDH activity, further contributing to impaired BCAA metabolism and altered energy homeostasis in the heart [134]. Figure 5 demonstrates in young hearts, efficient catabolism of BCAAs and glutamine through BCKDH fuels the tricarboxylic acid (TCA) cycle, enhancing ATP production, preserving mitochondrial integrity, and supporting optimal cardiac function. Elevated SIRT1 and PGC-1α levels further promote mitochondrial biogenesis and regulatory pathways, maintaining robust energy homeostasis. In contrast, aged hearts exhibit impaired BCAA catabolism due to reduced BCKDH activity, leading to the accumulation of BCKAs, decreased PP2Cm expression, and compromised mitochondrial complex I function. These changes result in reduced mitochondrial performance, increased ROS generation, and progressive mitochondrial damage, while downregulation of SIRT1 and PGC-1α exacerbates the decline in mitochondrial biogenesis and cardiac energy metabolism [135,136]. The upregulation of PP2Cm, have been shown to reverse mitochondrial deficits and improve cardiac contractile performance, likely through the activation of PGC-1α/SIRT1-mediated pathways [137]. Beyond BCAAs, other amino acids such as glycine, glutamine, and arginine also play critical roles in maintaining mitochondrial function. Glycine contributes to glutathione synthesis, mitigates oxidative stress, and enhances mitochondrial efficiency in cardiomyocytes [138]. Glutamine functions as both a respiratory substrate, through its conversion to α-ketoglutarate, and a nitrogen donor, supporting TCA cycle activity and antioxidant defenses. Meanwhile, arginine plays a dual role by replenishing TCA intermediates via the urea cycle and promoting nitric oxide production [139]. These findings highlight the dual role of BCAAs and other amino acids not only as metabolic substrates fueling ATP synthesis, but also as critical signaling molecules that regulate mitochondrial biogenesis, oxidative stress responses, and overall cardiomyocyte energy homeostasis.

### 8.2. Enhancing Mitophagy

Mitophagy enhances mitochondrial quality through the removal of old mitochondria. Induction of mitophagy is a therapeutic approach with potential that has the capacity to enhance mitochondrial function during senescence. Small molecules such as urolithin A and spermidine were reported to induce mitophagy and increase lifespan in model organisms [140,141]. Urolithin A, a dietary ellagitannin metabolite, improves mitochondrial function by inducing mitophagy in aged muscle cells and improving endurance capacity and muscle strength in animal models. Spermidine, a dietary polyamine molecule, induces autophagy and mitophagy and exerts neuroprotection against cognitive dysfunction and cardiomyopathy induced by aging [142]. NAD^+^ precursors like nicotinamide riboside (NR) also induce mitophagy via induction of sirtuin-1 (SIRT1) and PGC-1α pathways [143]. New small compounds such as VL-004 induce mitophagy and induce lifespan extension in C. elegans through dct-1, the worm homolog of mammalian mitophagy receptor BNIP3 and BNIP3L/NIX [144]. Most importantly of all, VL-004 activity is absent in dendritic cell kinase mutant dct-1, which confirms the central importance that mitophagy performs. Although these compounds are highly promising, mammalian aging potency must be demonstrated by subsequent research.

### 8.3. Promoting Mitochondrial Biogenesis

Mitochondrial biogenesis is regulated through transcriptional coactivators such as PGC-1α, which integrate nuclear and mitochondrial gene expression in energy metabolism [145]. Therapeutic interventions for inducing biogenesis are aimed at replenishing mitochondrial pools and augmenting cellular bioenergetics. Exercise and caloric restriction stimulate PGC-1α and downstream targets, increasing mitochondrial number and activity in elderly tissues [146,147]. AMPK and sirtuin pharmacological activators also produce the same effects. The SIRT1 activator resveratrol increases mitochondrial biogenesis and metabolic efficiency in aged mice [148]. AMPK activators like AICAR also stimulate mitochondrial gene expression and increase muscle function [149]. The combination of biogenesis enhancers and mitophagy inducers would exhibit synergistic action in restoring mitochondrial homeostasis in aging cells

### 8.4. Modulating Mitochondrial Dynamics

Mitochondrial fission and fusion maintain organelle shape and integrity, allowing adaptation of metabolic demand and segregation of the defective organelles. Increased imbalance with age towards increased fission results in damaged mitochondria, impaired respiration, and increased ROS [150]. Dynamin-related protein 1 fission mediators/inhibitors such as Mdivi-1 inhibit mitochondrial fragmentation and restore age-related dysfunction in preclinical models [151]. Induction of increased fusion through the overexpression of mitofusins (Mfn1/2) or optic atrophy 1 also increases mitochondrial respiration and stress resistance [152]. Mitochondrial dynamics has been implicated as a method of maintaining mitochondrial integrity and retarding aging phenotypes.

### 8.5. Mitochondria-Targeted Antioxidants

Preclinical models have tested several of the therapeutic approaches against mitochondrial dysfunction with emphasis on induction of mitophagy, antioxidant defense, activation of mitochondrial biogenesis, and modulation of mitochondrial dynamics. Classical antioxidants have been of limited effectiveness in aging because they are inefficient at targeting mitochondria and possessing bioavailability. In an attempt to bypass this, mitochondria-targeted antioxidants have been designed to selectively accumulate inside the mitochondria and mop up ROS at the site of production [153]. MitoQ treatment in old mice reduced oxidative damage, improved endothelial function, and reduced vascular stiffening, all predictors of cardiovascular aging [154]. Similarly, MitoTEMPO reduced mitochondrial superoxide and improved systolic and diastolic cardiac function in older rodents [155]. Mitochondria-directed tetrapeptide SS-31 (elamipretide) was also effective in reducing oxidative stress and restoring cardiac hypertrophy after chronic treatment [156]. These findings illustrate that mitochondrial antioxidants rejuvenate redox homeostasis and augment mitochondrial bioenergetics in aged tissues.

### 8.6. Small-Molecule Mitochondrial Inhibitors

In the aging heart, mitochondria are both the primary energy source and a major site of ROS generation. In aged cardiomyocytes, partial inhibition of specific ETC complexes mimics features of mitochondrial aging, including reduced ATP production, increased ROS leakage, and altered redox signaling. For example, low-dose rotenone (Complex I inhibitor) or antimycin A (Complex III inhibitor) can exacerbate oxidative damage and activate maladaptive stress responses resembling those seen in senescent myocardium [157,158]. Similarly, oligomycin-mediated ATP synthase inhibition highlights the heart’s declining capacity to maintain energy homeostasis under metabolic stress with age [159]. These inhibitors have also been instrumental in uncovering the role of mitochondrial dysfunction in key aging hallmarks, impaired mitophagy, altered mitochondrial dynamics, and compromised calcium handling which together accelerate diastolic dysfunction, fibrosis, and reduced stress resilience in elderly hearts. Interestingly, in some experimental contexts, transient or partial mitochondrial inhibition can activate adaptive stress responses, such as mild uncoupling via FCCP, which triggers mitochondrial biogenesis [160].

Beyond their role as research tools, mitochondrial inhibitors may have therapeutic potential in modulating cardiac metabolism and stress responses. For instance, compounds such as CCG-1423, which impair mitochondrial function in cardiomyoblasts, could potentially be leveraged to target hyperactive mitochondrial metabolism in aging hearts or pathological states characterized by oxidative stress and heightened energetic demand [161,162]. Thus, while chronic or excessive mitochondrial inhibition is generally detrimental, carefully controlled modulation of mitochondrial activity may offer avenues to ameliorate age-related cardiac dysfunction, highlighting both investigative and translational relevance of these compounds.

### 8.7. Emerging Therapies: Mitochondrial Transplantation and Gene Editing

**Figure 6:**
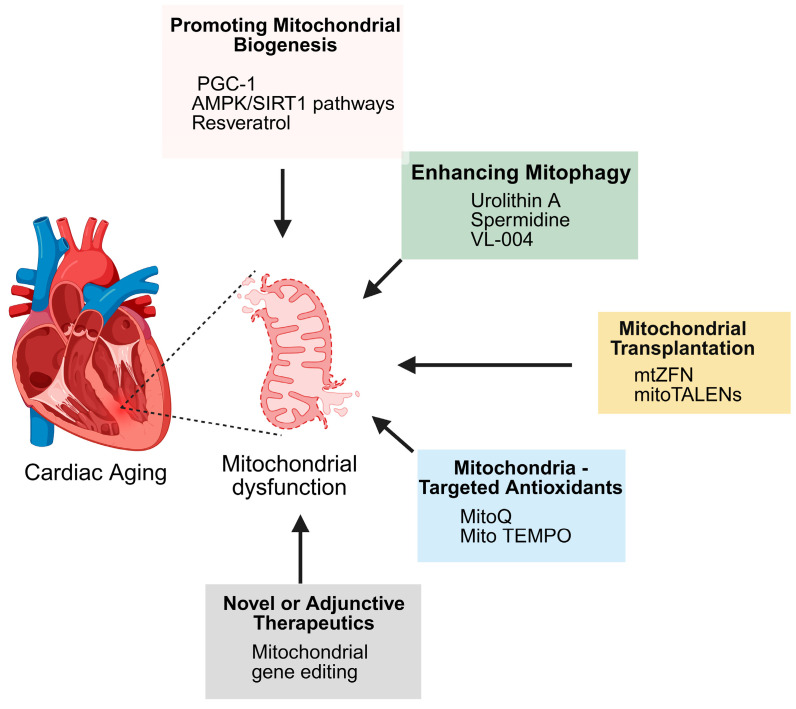
Therapeutic strategies targeting mitochondrial dysfunction in cardiac aging. Cardiac aging is associated with mitochondrial dysfunction, which can be addressed through multiple approaches: promoting mitochondrial biogenesis (via PGC-1, AMPK/SIRT1 pathways, resveratrol), enhancing mitophagy (urolithin A, spermidine, VL-004), mitochondrial transplantation (mtZFN, mitoTALENs), mitochondria-targeted antioxidants (MitoQ, Mito TEMPO), and novel or adjunctive therapies such as mitochondrial gene editing.

New modalities like mitochondrial transplantation and gene editing address substitution or repair of defective mitochondria. Mitochondrial transplantation refers to infusion of healthy mitochondria into defective cells, rejuvenating bioenergetics [163]. Experimental as it sounds, it holds promise in cardiac ischemia–reperfusion injury and may be employed in age-related mitochondrial deficiency. Gene editing tools such as mitochondrial-targeted zinc finger nucleases (mitoZFNs) and mitoTALENs as well as mitochondria-specific restriction enzymes are being developed to selectively eliminate mutant mtDNA [164,165]. A summary of these different therapeutic approaches is presented in Figure 6.

### 8.8. Challenges and Future Perspectives

Despite notable advances, the clinical translation of mitochondrial-targeted therapies remains challenging. Pronounced mitochondrial heterogeneity across tissues and between individuals complicates the development of uniform treatment strategies [166]. Rigorous evaluation of safety and off-target effects is essential, especially for long-term interventions. Robust biomarkers for mitochondrial health in cardiac disease such as GDF-15 and FGF-21 (elevated under stress), circulating mitochondrial DNA (mtDNA), acylcarnitines reflecting metabolic activity, and markers of oxidative stress like methylmalonic acid, are critical for proper patient stratification and monitoring of therapeutic efficacy [167,168]. Emerging tools, such as high-resolution mitochondrial imaging, metabolomics, and circulating mitochondrial DNA analysis, offer promise in closing this gap [169,170]. Furthermore, many interventions, such as antioxidants, modulators of mitochondrial dynamics, or enhancers of mitophagy, have shown efficacy in preclinical models but often demonstrate limited effectiveness in humans due to poor tissue specificity, short half-life, and off-target effects. Additionally, most approaches target a single pathway, whereas mitochondrial dysfunction in aging hearts is multifactorial, suggesting that combination therapies or more precise, targeted strategies may be necessary to achieve meaningful clinical benefits. Future work must investigate further the mechanisms of tissue-specific mitochondrial aging, explore synergistic use of mitochondrial therapies with systemic anti-aging approaches (e.g., senolytics, metabolic modulators), and conduct well-designed clinical trials to establish efficacy and safety.

## 9. Conclusions

Progressive alterations in mitochondrial morphology, impaired oxidative phosphorylation, disrupted dynamics, defective quality control, and accumulated mtDNA damage collectively erode the heart’s bioenergetic capacity and resilience. These changes not only limit ATP production but also elevate ROS generation, fueling oxidative stress, inflammation, and multimodal regulated cell death pathways that accelerate cardiomyocyte loss and fibrosis. Mitochondrial sirtuins, amino acid metabolism, and redox regulation emerge as central modulators of this process, highlighting potential molecular targets for intervention. Therapeutic strategies aimed at restoring mitochondrial health, including enhancing mitophagy, promoting biogenesis, modulating dynamics, targeting redox balance, and optimizing amino acid metabolism, show considerable promise in delaying or reversing cardiac aging. However, challenges such as mitochondrial heterogeneity, tissue-specific responses, long-term safety, and the need for robust biomarkers remain substantial barriers to clinical translation. Future research integrating mechanistic insights with innovative delivery platforms and personalized therapeutic approaches will be essential for transforming these experimental advances into viable interventions, ultimately aiming to extend cardiovascular health span and resilience in the aging population.

## Data Availability

No new data were created or analyzed in this study.

## References

[1] Ribeiro A.S.F., Zerolo B.E., López-Espuela F., Sánchez R., Fernandes V.S. (2023). Cardiac System during the Aging Process. Aging Dis..

[2] Tenchov R., Sasso J.M., Wang X., Zhou Q.A. (2024). Aging Hallmarks and Progression and Age-Related Diseases: A Landscape View of Research Advancement. ACS Chem. Neurosci..

[3] Boyman L., Karbowski M., Lederer W.J. (2020). Regulation of Mitochondrial ATP Production: Ca^2+^ Signaling and Quality Control. Trends Mol. Med..

[4] Popoiu T.-A., Maack C., Bertero E. (2023). Mitochondrial Calcium Signaling and Redox Homeostasis in Cardiac Health and Disease. Front. Mol. Med..

[5] Tocchi A., Quarles E.K., Basisty N., Gitari L., Rabinovitch P.S. (2015). Mitochondrial Dysfunction in Cardiac Aging. Biochim. Biophys. Acta.

[6] Wei P., Zhang X., Yan C., Sun S., Chen Z., Lin F. (2025). Mitochondrial dysfunction and aging: Multidimensional mechanisms and therapeutic strategies. Biogerontology.

[7] Quiles J.M., Gustafsson Å.B. (2022). The Role of Mitochondrial Fission in Cardiovascular Health and Disease. Nat. Rev. Cardiol..

[8] Liang W.J., Gustafsson Å.B. (2020). The Aging Heart: Mitophagy at the Center of Rejuvenation. Front. Cardiovasc. Med..

[9] Ding W., Chen J., Zhao L., Wu S., Chen X., Chen H. (2024). Mitochondrial DNA Leakage Triggers Inflammation in Age-Related Cardiovascular Diseases. Front. Cell Dev. Biol..

[10] Sagar S., Gustafsson A.B. (2023). Cardiovascular Aging: The Mitochondrial Influence. J. Cardiovasc. Aging.

[11] Sheng S.-Y., Li J.-M., Hu X.-Y., Wang Y. (2023). Regulated Cell Death Pathways in Cardiomyopathy. Acta Pharmacol. Sin..

[12] Yu Y., Yan Y., Niu F., Wang Y., Chen X., Su G., Liu Y., Zhao X., Qian L., Liu P. (2021). Ferroptosis: A Cell Death Connecting Oxidative Stress, Inflammation and Cardiovascular Diseases. Cell Death Discov..

[13] Lombard D.B., Tishkoff D.X., Bao J. (2011). Mitochondrial Sirtuins in the Regulation of Mitochondrial Activity and Metabolic Adaptation. Handb. Exp. Pharmacol..

[14] Li Q., Hoppe T. (2023). Role of Amino Acid Metabolism in Mitochondrial Homeostasis. Front. Cell Dev. Biol..

[15] Zhu X., Shen W., Yao K., Wang H., Liu B., Li T., Song L., Diao D., Mao G., Huang P. (2019). Fine-Tuning of PGC1α Expression Regulates Cardiac Function and Longevity. Circ. Res..

[16] Wang S., Long H., Hou L., Feng B., Ma Z., Wu Y., Zeng Y., Cai J., Zhang D.-W., Zhao G. (2023). The Mitophagy Pathway and Its Implications in Human Diseases. Signal Transduct. Target. Ther..

[17] Hernández-Camacho J.D., Fernández-Ayala D.J.M., Vicente-García C., Navas-Enamorado I., López-Lluch G., Oliva C., Artuch R., Garcia-Villoria J., Ribes A., de Cabo R. (2022). Calorie Restriction Rescues Mitochondrial Dysfunction in Adck2-Deficient Skeletal Muscle. Front. Physiol..

[18] Ren J., Zhang Y. (2018). Targeting Autophagy in Aging and Aging-Related Cardiovascular Diseases. Trends Pharmacol. Sci..

[19] Chen L., Chen M., Yang X., Hu Y., Qiu C., Fu Y., Lan X., Luo G., Liu Q., Liu M. (2025). Energy Metabolism in Cardiovascular Diseases: Unlocking the Hidden Powerhouse of Cardiac Pathophysiology. Front. Endocrinol..

[20] Sun Q., Karwi Q.G., Wong N., Lopaschuk G.D. (2024). Advances in Myocardial Energy Metabolism: Metabolic Remodelling in Heart Failure and Beyond. Cardiovasc. Res..

[21] Aon M.A., Cortassa S. (2012). Mitochondrial Network Energetics in the Heart. Wiley Interdiscip. Rev. Syst. Biol. Med..

[22] Hinton A., Claypool S.M., Neikirk K., Senoo N., Wanjalla C.N., Kirabo A., Williams C.R. (2024). Mitochondrial Structure and Function in Human Heart Failure. Circ. Res..

[23] Mone P., Agyapong E.D., Morciano G., Jankauskas S.S., De Luca A., Varzideh F., Pinton P., Santulli G. (2024). Dysfunctional mitochondria elicit bioenergetic decline in the aged heart. J. Cardiovasc. Aging.

[24] Boengler K., Kosiol M., Mayr M., Schulz R., Rohrbach S. (2017). Mitochondria and Ageing: Role in Heart, Skeletal Muscle and Adipose Tissue. J. Cachexia Sarcopenia Muscle.

[25] Nakano K., Yamamoto M., Yamada Y., Nakatsukasa T., Kawamatsu N., Sato K., Machino-Ohtsuka T., Murakoshi N., Ishizu T. (2024). Mitochondrial Structural Abnormalities and Cardiac Reverse Remodeling in Patients With Systolic Dysfunction. Circ. J..

[26] Huang C., Deng K., Wu M. (2023). Mitochondrial Cristae in Health and Disease. Int. J. Biol. Macromol..

[27] Riva A., Tandler B., Lesnefsky E.J., Conti G., Loffredo F., Vazquez E., Hoppel C.L. (2006). Structure of cristae in cardiac mitochondria of aged rat. Mech. Ageing Dev..

[28] Collins H.E., Kane M.S., Litovsky S.H., Darley-Usmar V.M., Young M.E., Chatham J.C., Zhang J. (2021). Mitochondrial Morphology and Mitophagy in Heart Diseases: Qualitative and Quantitative Analyses Using Transmission Electron Microscopy. Front. Aging.

[29] Chen Q., Samidurai A., Thompson J., Hu Y., Das A., Willard B., Lesnefsky E.J. (2020). Endoplasmic Reticulum Stress-Mediated Mitochondrial Dysfunction in Aged Hearts. Biochim. Biophys. Acta Mol. Basis Dis..

[30] Xu X., Pang Y., Fan X. (2025). Mitochondria in Oxidative Stress, Inflammation and Aging: From Mechanisms to Therapeutic Advances. Signal Transduct. Target. Ther..

[31] Miró O., Casademont J., Casals E., Perea M., Urbano-Márquez A., Rustin P., Cardellach F. (2000). Aging is associated with increased lipid peroxidation in human hearts, but not with mitochondrial respiratory chain enzyme defects. Cardiovasc. Res..

[32] Chen Q., Thompson J., Hu Y., Wang H., Slotabec L., Nguyen J.D., Rouhi N., Li J., Lesnefsky E.J. (2024). High-dose metformin treatment to inhibit complex I during early reperfusion protects the aged mouse heart via decreased mitochondrial permeability transition pore opening. J. Pharmacol. Exp. Ther..

[33] Chen Q., Thompson J., Hu Y., Lesnefsky E.J. (2024). Aging-Induced Mitochondrial Dysfunction: Two Distinct Populations of Mitochondria versus a Combined Population. Am. J. Physiol. Heart Circ. Physiol..

[34] Dai D.-F., Chen T., Wanagat J., Laflamme M., Marcinek D.J., Emond M.J., Ngo C.P., Prolla T.A., Rabinovitch P.S. (2010). Age-Dependent Cardiomyopathy in Mitochondrial Mutator Mice Is Attenuated by Overexpression of Catalase Targeted to Mitochondria. Aging Cell.

[35] Sun N., Youle R.J., Finkel T. (2016). The Mitochondrial Basis of Aging. Mol. Cell.

[36] Gabillard-Lefort C., Thibault T., Lenaers G., Wiesner R.J., Mialet-Perez J., Baris O.R. (2025). Heart of the Matter: Mitochondrial Dynamics and Genome Alterations in Cardiac Aging. Mech. Ageing Dev..

[37] Tezze C., Romanello V., Desbats M.A., Fadini G.P., Albiero M., Favaro G., Ciciliot S., Soriano M.E., Morbidoni V., Cerqua C. (2017). Age-Associated Loss of OPA1 in Muscle Impacts Muscle Mass, Metabolic Homeostasis, Systemic Inflammation, and Epithelial Senescence. Cell Metab..

[38] Sebastián D., Sorianello E., Segalés J., Irazoki A., Ruiz-Bonilla V., Sala D., Planet E., Berenguer-Llergo A., Muñoz J.P., Sánchez-Feutrie M. (2016). Mfn2 Deficiency Links Age-Related Sarcopenia and Impaired Autophagy to Activation of an Adaptive Mitophagy Pathway. EMBO J..

[39] Zhang H., Yu F., Tian Z., Jia D. (2025). Cardiolipin Remodeling in Cardiovascular Diseases: Implication for Mitochondrial Dysfunction. Acta Physiol..

[40] Jin J.-Y., Wei X.-X., Zhi X.-L., Wang X.-H., Meng D. (2021). Drp1-Dependent Mitochondrial Fission in Cardiovascular Disease. Acta Pharmacol. Sin..

[41] Liu Y.J., McIntyre R.L., Janssens G.E., Houtkooper R.H. (2020). Mitochondrial Fission and Fusion: A Dynamic Role in Aging and Potential Target for Age-Related Disease. Mech. Ageing Dev..

[42] Liu B.-H., Xu C.-Z., Liu Y., Lu Z.-L., Fu T.-L., Li G.-R., Deng Y., Luo G.-Q., Ding S., Li N. (2024). Mitochondrial Quality Control in Human Health and Disease. Mil. Med. Res..

[43] Zha Z., Wang J., Wang X., Lu M., Guo Y. (2017). Involvement of PINK1/Parkin-Mediated Mitophagy in AGE-Induced Cardiomyocyte Aging. Int. J. Cardiol..

[44] Qi X.-M., Qiao Y.-B., Zhang Y.-L., Wang A.-C., Ren J.-H., Wei H.-Z., Li Q.-S. (2023). PGC-1α/NRF1-Dependent Cardiac Mitochondrial Biogenesis: A Druggable Pathway of Calycosin against Triptolide Cardiotoxicity. Food Chem. Toxicol..

[45] Wang Y., Zhang X., Wen Y., Li S., Lu X., Xu R., Li C. (2021). Endoplasmic Reticulum-Mitochondria Contacts: A Potential Therapy Target for Cardiovascular Remodeling-Associated Diseases. Front. Cell Dev. Biol..

[46] Chen Q., Thompson J., Hu Y., Lesnefsky E.J. (2022). Reversing Mitochondrial Defects in Aged Hearts: Role of Mitochondrial Calpain Activation. Am. J. Physiol. Cell Physiol..

[47] Mendoza A., Karch J. (2022). Keeping the Beat against Time: Mitochondrial Fitness in the Aging Heart. Front. Aging.

[48] Kaludercic N., Mialet-Perez J., Paolocci N., Parini A., Di Lisa F. (2014). Monoamine Oxidases as Sources of Oxidants in the Heart. J. Mol. Cell Cardiol..

[49] Ungvari Z., Kaley G., de Cabo R., Sonntag W.E., Csiszar A. (2010). Mechanisms of Vascular Aging: New Perspectives. J. Gerontol. A Biol. Sci. Med. Sci..

[50] Ungvari Z., Orosz Z., Labinskyy N., Rivera A., Xiangmin Z., Smith K., Csiszar A. (2007). Increased Mitochondrial H_2_O_2_ Production Promotes Endothelial NF-κB Activation in Aged Rat Arteries. Am. J. Physiol. Heart Circ. Physiol..

[51] Peoples J.N., Saraf A., Ghazal N., Pham T.T., Kwong J.Q. (2019). Mitochondrial Dysfunction and Oxidative Stress in Heart Disease. Exp. Mol. Med..

[52] Koutouroushis C., Sarkar O. (2021). Role of Autophagy in Cardiovascular Disease and Aging. Cureus.

[53] Green A.P., Klimm F., Marshall A.S., Leetmaa R., Aryaman J., Gómez-Durán A., Chinnery P.F., Jones N.S. (2025). Cryptic Mitochondrial DNA Mutations Coincide with Mid-Late Life and Are Pathophysiologically Informative in Single Cells across Tissues and Species. Nat. Commun..

[54] Luan Y., Zhu X., Jiao Y., Liu H., Huang Z., Pei J., Xu Y., Yang Y., Ren K. (2024). Cardiac Cell Senescence: Molecular Mechanisms, Key Proteins and Therapeutic Targets. Cell Death Discov..

[55] Patyal P., Azhar G., Zhang X., Verma A., Wei J.Y. (2025). Cardiac-Specific Overexpression of Serum Response Factor Regulates Age-Associated Decline in Mitochondrial Function. Geroscience.

[56] Jeon S.-M. (2016). Regulation and Function of AMPK in Physiology and Diseases. Exp. Mol. Med..

[57] Borodkina A., Shatrova A., Abushik P., Nikolsky N., Burova E. (2014). Interaction between ROS Dependent DNA Damage, Mitochondria and P38 MAPK Underlies Senescence of Human Adult Stem Cells. Aging.

[58] Zhang X., Williams E.D., Azhar G., Rogers S.C., Wei J.Y. (2016). Does P49/STRAP, a SRF-Binding Protein (SRFBP1), Modulate Cardiac Mitochondrial Function in Aging?. Exp. Gerontol..

[59] Zhang X., Azhar G., Zhong Y., Wei J.Y. (2004). Identification of a Novel Serum Response Factor Cofactor in Cardiac Gene Regulation. J. Biol. Chem..

[60] Krošel M., Gabathuler M., Moser L., Maciukiewicz M., Züllig T., Seifritz T., Tomšič M., Distler O., Ospelt C., Klein K. (2023). The Histone Acetyl Transferases CBP and P300 Regulate Stress Response Pathways in Synovial Fibroblasts at Transcriptional and Functional Levels. Sci. Rep..

[61] Dabravolski S.A., Nikiforov N.G., Zhuravlev A.D., Orekhov N.A., Grechko A.V., Orekhov A.N. (2022). Role of the mtDNA Mutations and Mitophagy in Inflammaging. Int. J. Mol. Sci..

[62] Shirakabe A., Ikeda Y., Sciarretta S., Zablocki D.K., Sadoshima J. (2016). Aging and Autophagy in the Heart. Circ. Res..

[63] Liu X., Si W., He L., Yang J., Peng Y., Ren J., Liu X., Jin T., Yu H., Zhang Z. (2021). The Existence of a Nonclassical TCA Cycle in the Nucleus That Wires the Metabolic-Epigenetic Circuitry. Signal Transduct. Target. Ther..

[64] Zhang X., Zhang F., Zeng Y., Li A., Yan J., Li P., Qin K., Zhang T., Huang J., Zhao M. (2025). Mitochondrial Dysfunction-Mediated Metabolic Remodeling of TCA Cycle Promotes Parkinson’s Disease through Inhibition of H3K4me3 Demethylation. Cell Death Discov..

[65] Wen H., Deng H., Li B., Chen J., Zhu J., Zhang X., Yoshida S., Zhou Y. (2025). Mitochondrial Diseases: From Molecular Mechanisms to Therapeutic Advances. Signal Transduct. Target. Ther..

[66] Tait S.W.G., Green D.R. (2013). Mitochondrial Regulation of Cell Death. Cold Spring Harb. Perspect. Biol..

[67] Tian C., Liu Y., Li Z., Zhu P., Zhao M. (2022). Mitochondria Related Cell Death Modalities and Disease. Front. Cell Dev. Biol..

[68] Picca A., Mankowski R.T., Burman J.L., Donisi L., Kim J.-S., Marzetti E., Leeuwenburgh C. (2018). Mitochondrial Quality Control Mechanisms as Molecular Targets in Cardiac Ageing. Nat. Rev. Cardiol..

[69] Mustafa M., Ahmad R., Tantry I.Q., Ahmad W., Siddiqui S., Alam M., Abbas K., Moinuddin, Hassan M.I., Habib S. (2024). Apoptosis: A Comprehensive Overview of Signaling Pathways, Morphological Changes, and Physiological Significance and Therapeutic Implications. Cells.

[70] Nguyen T.T., Wei S., Nguyen T.H., Jo Y., Zhang Y., Park W., Gariani K., Oh C.-M., Kim H.H., Ha K.-T. (2023). Mitochondria-Associated Programmed Cell Death as a Therapeutic Target for Age-Related Disease. Exp. Mol. Med..

[71] Kim H.-E., Du F., Fang M., Wang X. (2005). Formation of Apoptosome Is Initiated by Cytochrome C-Induced dATP Hydrolysis and Subsequent Nucleotide Exchange on Apaf-1. Proc. Natl. Acad. Sci. USA.

[72] Kwak H.-B. (2013). Effects of Aging and Exercise Training on Apoptosis in the Heart. J. Exerc. Rehabil..

[73] Kale J., Osterlund E.J., Andrews D.W. (2018). BCL-2 Family Proteins: Changing Partners in the Dance towards Death. Cell Death Differ..

[74] Roufayel R., Younes K., Al-Sabi A., Murshid N. (2022). BH3-Only Proteins Noxa and Puma Are Key Regulators of Induced Apoptosis. Life.

[75] Liu L., Azhar G., Gao W., Zhang X., Wei J.Y. (1998). Bcl-2 and Bax Expression in Adult Rat Hearts after Coronary Occlusion: Age-Associated Differences. Am. J. Physiol..

[76] Azhar G., Liu L., Zhang X., Wei J.Y. (1999). Influence of Age on Hypoxia/Reoxygenation-Induced DNA Fragmentation and Bcl-2, Bcl-Xl, Bax and Fas in the Rat Heart and Brain. Mech. Ageing Dev..

[77] Borrás C., Mas-Bargues C., Román-Domínguez A., Sanz-Ros J., Gimeno-Mallench L., Inglés M., Gambini J., Viña J. (2020). BCL-xL, a Mitochondrial Protein Involved in Successful Aging: From C. Elegans to Human Centenarians. Int. J. Mol. Sci..

[78] Summer S., Borrell-Pages M., Bruno R.-M., Climie R.E., Dipla K., Dogan A., Eruslanova K., Fraenkel E., Mattace-Raso F., Pugh C.J.A. (2025). Centenarians-the Way to Healthy Vascular Ageing and Longevity: A Review from VascAgeNet. Geroscience.

[79] Guerrache A., Micheau O. (2024). TNF-Related Apoptosis-Inducing Ligand: Non-Apoptotic Signalling. Cells.

[80] Hung C.-L., Chang H.-H., Lee S.W., Chiang Y.-W. (2021). Stepwise Activation of the Pro-Apoptotic Protein Bid at Mitochondrial Membranes. Cell Death Differ..

[81] DeRoo E., Zhou T., Liu B. (2020). The Role of RIPK1 and RIPK3 in Cardiovascular Disease. Int. J. Mol. Sci..

[82] Chen B., Xie K., Zhang J., Yang L., Zhou H., Zhang L., Peng R. (2023). Comprehensive Analysis of Mitochondrial Dysfunction and Necroptosis in Intracranial Aneurysms from the Perspective of Predictive, Preventative, and Personalized Medicine. Apoptosis.

[83] Zhang X., Gao Y., Zhang S., Wang Y., Pei X., Chen Y., Zhang J., Zhang Y., Du Y., Hao S. (2025). Mitochondrial Dysfunction in the Regulation of Aging and Aging-Related Diseases. Cell Commun. Signal..

[84] Yang X., Li G., Lou P., Zhang M., Yao K., Xiao J., Chen Y., Xu J., Tian S., Deng M. (2024). Excessive Nucleic Acid R-Loops Induce Mitochondria-Dependent Epithelial Cell Necroptosis and Drive Spontaneous Intestinal Inflammation. Proc. Natl. Acad. Sci. USA.

[85] Verma A., Azhar G., Zhang X., Patyal P., Kc G., Sharma S., Che Y., Wei J.Y. (2023). *P. gingivalis*-LPS Induces Mitochondrial Dysfunction Mediated by Neuroinflammation through Oxidative Stress. Int. J. Mol. Sci..

[86] Verma A., Azhar G., Patyal P., Zhang W., Zhang X., Wei J.Y. (2024). Proteomic Analysis of P. Gingivalis-Lipopolysaccharide Induced Neuroinflammation in SH-SY5Y and HMC3 Cells. Geroscience.

[87] Verma A., Azhar G., Patyal P., Zhang X., Wei J.Y. (2025). Porphyromonas Gingivalis-Lipopolysaccharide Induced Caspase-4 Dependent Noncanonical Inflammasome Activation Drives Alzheimer’s Disease Pathologies. Cells.

[88] Azhar G., Nagano K., Patyal P., Zhang X., Verma A., Wei J.Y. (2024). Deletion of Interleukin-1β Converting Enzyme Alters Mouse Cardiac Structure and Function. Biology.

[89] Evavold C.L., Hafner-Bratkovič I., Devant P., D’Andrea J.M., Ngwa E.M., Boršić E., Doench J.G., LaFleur M.W., Sharpe A.H., Thiagarajah J.R. (2021). Control of Gasdermin D Oligomerization and Pyroptosis by the Ragulator-Rag-mTORC1 Pathway. Cell.

[90] Miao R., Jiang C., Chang W.Y., Zhang H., An J., Ho F., Chen P., Zhang H., Junqueira C., Amgalan D. (2023). Gasdermin D Permeabilization of Mitochondrial Inner and Outer Membranes Accelerates and Enhances Pyroptosis. Immunity.

[91] Li J., Jia Y.-C., Ding Y.-X., Bai J., Cao F., Li F. (2023). The Crosstalk between Ferroptosis and Mitochondrial Dynamic Regulatory Networks. Int. J. Biol. Sci..

[92] Bi Y., Liu S., Qin X., Abudureyimu M., Wang L., Zou R., Ajoolabady A., Zhang W., Peng H., Ren J. (2024). FUNDC1 Interacts with GPx4 to Govern Hepatic Ferroptosis and Fibrotic Injury through a Mitophagy-Dependent Manner. J. Adv. Res..

[93] Fratta Pasini A.M., Stranieri C., Busti F., Di Leo E.G., Girelli D., Cominacini L. (2023). New Insights into the Role of Ferroptosis in Cardiovascular Diseases. Cells.

[94] Wang K., Chen X.-Z., Wang Y.-H., Cheng X.-L., Zhao Y., Zhou L.-Y., Wang K. (2022). Emerging Roles of Ferroptosis in Cardiovascular Diseases. Cell Death Discov..

[95] Stockwell B.R., Jiang X., Gu W. (2020). Emerging Mechanisms and Disease Relevance of Ferroptosis. Trends Cell Biol..

[96] Wang J., Chen Z., Shang H., Guo J. (2025). The Molecular Mechanisms of Cuproptosis and Its Relevance to Atherosclerosis. Biomol. Biomed..

[97] Chen L., Wei J., Deng G., Xu G. (2025). Disulfidptosis-Related Gene in Acute Myocardial Infarction and Its Diagnostic Value and Functions Based on Bioinformatics Analysis and Machine Learning. Front. Cardiovasc. Med..

[98] Carollo C., Sorce A., Cirafici E., Mulè G., Caimi G. (2025). Sirtuins and Resveratrol in Cardiorenal Diseases: A Narrative Review of Mechanisms and Therapeutic Potential. Nutrients.

[99] Ding Y.-N., Wang H.-Y., Chen X.-F., Tang X., Chen H.-Z. (2025). Roles of Sirtuins in Cardiovascular Diseases: Mechanisms and Therapeutics. Circ. Res..

[100] Patyal P., Ameer F.S., Verma A., Zhang X., Azhar G., Shrivastava J., Sharma S., Zhang R., Wei J.Y. (2024). The Role of Sirtuin-1 Isoforms in Regulating Mitochondrial Function. Curr. Issues Mol. Biol..

[101] Perico L., Morigi M., Pezzotta A., Corna D., Brizi V., Conti S., Zanchi C., Sangalli F., Trionfini P., Buttò S. (2021). Post-Translational Modifications by SIRT3 de-2-Hydroxyisobutyrylase Activity Regulate Glycolysis and Enable Nephrogenesis. Sci. Rep..

[102] Mathias R.A., Greco T.M., Oberstein A., Budayeva H.G., Chakrabarti R., Rowland E.A., Kang Y., Shenk T., Cristea I.M. (2014). Sirtuin 4 Is a Lipoamidase Regulating Pyruvate Dehydrogenase Complex Activity. Cell.

[103] Du J., Zhou Y., Su X., Yu J.J., Khan S., Jiang H., Kim J., Woo J., Kim J.H., Choi B.H. (2011). Sirt5 Is a NAD-Dependent Protein Lysine Demalonylase and Desuccinylase. Science.

[104] Sidorova-Darmos E., Sommer R., Eubanks J.H. (2018). The Role of SIRT3 in the Brain Under Physiological and Pathological Conditions. Front. Cell Neurosci..

[105] Hafner A.V., Dai J., Gomes A.P., Xiao C.-Y., Palmeira C.M., Rosenzweig A., Sinclair D.A. (2010). Regulation of the mPTP by SIRT3-Mediated Deacetylation of CypD at Lysine 166 Suppresses Age-Related Cardiac Hypertrophy. Aging.

[106] Porter G.A., Urciuoli W.R., Brookes P.S., Nadtochiy S.M. (2014). SIRT3 Deficiency Exacerbates Ischemia-Reperfusion Injury: Implication for Aged Hearts. Am. J. Physiol. Heart Circ. Physiol..

[107] Ahn B.-H., Kim H.-S., Song S., Lee I.H., Liu J., Vassilopoulos A., Deng C.-X., Finkel T. (2008). A Role for the Mitochondrial Deacetylase Sirt3 in Regulating Energy Homeostasis. Proc. Natl. Acad. Sci. USA.

[108] Liu Y.-P., Wen R., Liu C.-F., Zhang T.-N., Yang N. (2023). Cellular and Molecular Biology of Sirtuins in Cardiovascular Disease. Biomed. Pharmacother..

[109] Wu Y.-T., Lee H.-C., Liao C.-C., Wei Y.-H. (2013). Regulation of Mitochondrial F_o_F_1_ATPase Activity by Sirt3-Catalyzed Deacetylation and Its Deficiency in Human Cells Harboring 4977bp Deletion of Mitochondrial DNA. Biochim. Biophys. Acta.

[110] Guo L., Yin A., Zhang Q., Zhong T., O’Rourke S.T., Sun C. (2017). Angiotensin-(1-7) Attenuates Angiotensin II-Induced Cardiac Hypertrophy via a Sirt3-Dependent Mechanism. Am. J. Physiol. Heart Circ. Physiol..

[111] Feng X., Wang Y., Chen W., Xu S., Li L., Geng Y., Shen A., Gao H., Zhang L., Liu S. (2020). SIRT3 Inhibits Cardiac Hypertrophy by Regulating PARP-1 Activity. Aging.

[112] Chen C.-J., Fu Y.-C., Yu W., Wang W. (2013). SIRT3 Protects Cardiomyocytes from Oxidative Stress-Mediated Cell Death by Activating NF-κB. Biochem. Biophys. Res. Commun..

[113] Farhadi Z., Esmailidehaj M., Masoumi S., Azizian H. (2025). Sirtuins as Endogenous Regulators of Cardiac Fibrosis: A Current Perspective. Cardiovasc. Toxicol..

[114] Hirschey M.D., Shimazu T., Goetzman E., Jing E., Schwer B., Lombard D.B., Grueter C.A., Harris C., Biddinger S., Ilkayeva O.R. (2010). SIRT3 Regulates Mitochondrial Fatty-Acid Oxidation by Reversible Enzyme Deacetylation. Nature.

[115] Shimazu T., Hirschey M.D., Hua L., Dittenhafer-Reed K.E., Schwer B., Lombard D.B., Li Y., Bunkenborg J., Alt F.W., Denu J.M. (2010). SIRT3 Deacetylates Mitochondrial 3-Hydroxy-3-Methylglutaryl CoA Synthase 2 and Regulates Ketone Body Production. Cell Metab..

[116] Min Z., Gao J., Yu Y. (2018). The Roles of Mitochondrial SIRT4 in Cellular Metabolism. Front. Endocrinol..

[117] Laurent G., German N.J., Saha A.K., de Boer V.C.J., Davies M., Koves T.R., Dephoure N., Fischer F., Boanca G., Vaitheesvaran B. (2013). SIRT4 Coordinates the Balance between Lipid Synthesis and Catabolism by Repressing Malonyl CoA Decarboxylase. Mol. Cell.

[118] Rardin M.J., He W., Nishida Y., Newman J.C., Carrico C., Danielson S.R., Guo A., Gut P., Sahu A.K., Li B. (2013). SIRT5 Regulates the Mitochondrial Lysine Succinylome and Metabolic Networks. Cell. Metab..

[119] Nakagawa T., Guarente L. (2009). Urea Cycle Regulation by Mitochondrial Sirtuin, SIRT5. Aging.

[120] Fabbrizi E., Fiorentino F., Carafa V., Altucci L., Mai A., Rotili D. (2023). Emerging Roles of SIRT5 in Metabolism, Cancer, and SARS-CoV-2 Infection. Cells.

[121] Shen R., Zhang Y. (2025). Relationship between Amino Acid Metabolism and Inflammation in Coronary Heart Disease (Review). Int. J. Mol. Med..

[122] Azhar G., Verma A., Zhang X., Pangle A., Patyal P., Zhang W., Che Y., Coker K., Wolfe R.R., Wei J.Y. (2023). Differential Plasma Protein Expression after Ingestion of Essential Amino Acid-Based Dietary Supplement Verses Whey Protein in Low Physical Functioning Older Adults. Geroscience.

[123] Azhar G., Verma A., Robeson M.S., Patyal P., Nookaew I., Sharma S., Pangle A., Che Y., Wolfe R.R., Wei J.Y. (2024). Short-Term Ingestion of Essential Amino Acid Based Nutritional Supplements or Whey Protein Improves the Physical Function of Older Adults Independently of Gut Microbiome. Mol. Nutr. Food Res..

[124] D’Antona G., Ragni M., Cardile A., Tedesco L., Dossena M., Bruttini F., Caliaro F., Corsetti G., Bottinelli R., Carruba M.O. (2010). Branched-Chain Amino Acid Supplementation Promotes Survival and Supports Cardiac and Skeletal Muscle Mitochondrial Biogenesis in Middle-Aged Mice. Cell Metab..

[125] Buondonno I., Sassi F., Carignano G., Dutto F., Ferreri C., Pili F.G., Massaia M., Nisoli E., Ruocco C., Porrino P. (2020). From Mitochondria to Healthy Aging: The Role of Branched-Chain Amino Acids Treatment: MATeR a Randomized Study. Clin. Nutr..

[126] Du C., Liu W.-J., Yang J., Zhao S.-S., Liu H.-X. (2022). The Role of Branched-Chain Amino Acids and Branched-Chain α-Keto Acid Dehydrogenase Kinase in Metabolic Disorders. Front. Nutr..

[127] Blair M.C., Neinast M.D., Arany Z. (2021). Whole-Body Metabolic Fate of Branched-Chain Amino Acids. Biochem. J..

[128] Bo T., Fujii J. (2024). Primary Roles of Branched Chain Amino Acids (BCAAs) and Their Metabolism in Physiology and Metabolic Disorders. Molecules.

[129] Sharma S., Zhang X., Azhar G., Patyal P., Verma A., Kc G., Wei J.Y. (2024). Valine Improves Mitochondrial Function and Protects against Oxidative Stress. Biosci. Biotechnol. Biochem..

[130] Sun H., Olson K.C., Gao C., Prosdocimo D.A., Zhou M., Wang Z., Jeyaraj D., Youn J.-Y., Ren S., Liu Y. (2016). Catabolic Defect of Branched-Chain Amino Acids Promotes Heart Failure. Circulation.

[131] Schwalb H., Kushnir T., Navon G., Yaroslavsky E., Borman J.B., Uretzky G. (1987). The Protective Effect of Enriched Branched Chain Amino Acid Formulation in the Ischemic Heart: A Phosphorous-31 Nuclear Magnetic Resonance Study. J. Mol. Cell. Cardiol..

[132] Hatazawa Y., Tadaishi M., Nagaike Y., Morita A., Ogawa Y., Ezaki O., Takai-Igarashi T., Kitaura Y., Shimomura Y., Kamei Y. (2014). PGC-1α-Mediated Branched-Chain Amino Acid Metabolism in the Skeletal Muscle. PLoS ONE.

[133] Hotta K., Taniguchi R., Nakayama H., Yamaguchi F., Sato Y. (2021). The Effects of an Oral Nutritional Supplement with Whey Peptides and Branched-Chain Amino Acids for Cardiac Rehabilitation of Patients with Chronic Heart Failure. Int. Heart J..

[134] Lu G., Sun H., She P., Youn J.Y., Warburton S., Ping P., Vondriska T.M., Cai H., Lynch C.J., Wang Y. (2009). Protein phosphatase 2Cm is a critical regulator of branched-chain amino acid catabolism in mice and cultured cells. J. Clin. Investig..

[135] Xiong Y., Jiang L., Li T. (2022). Aberrant Branched-Chain Amino Acid Catabolism in Cardiovascular Diseases. Front. Cardiovasc. Med..

[136] Murashige D., Jung J.W., Neinast M.D., Levin M.G., Chu Q., Lambert J.P., Garbincius J.F., Kim B., Hoshino A., Marti-Pamies I. (2022). Extra-Cardiac BCAA Catabolism Lowers Blood Pressure and Protects from Heart Failure. Cell Metab..

[137] Lian K., Guo X., Wang Q., Liu Y., Wang R.-T., Gao C., Li C.-Y., Li C.-X., Tao L. (2020). PP2Cm Overexpression Alleviates MI/R Injury Mediated by a BCAA Catabolism Defect and Oxidative Stress in Diabetic Mice. Eur. J. Pharmacol..

[138] Durante W. (2019). The Emerging Role of L-Glutamine in Cardiovascular Health and Disease. Nutrients.

[139] Fung T.S., Ryu K.W., Thompson C.B. (2025). Arginine: At the Crossroads of Nitrogen Metabolism. EMBO J..

[140] Ryu D., Mouchiroud L., Andreux P.A., Katsyuba E., Moullan N., Nicolet-Dit-Félix A.A., Williams E.G., Jha P., Lo Sasso G., Huzard D. (2016). Urolithin A Induces Mitophagy and Prolongs Lifespan in C. Elegans and Increases Muscle Function in Rodents. Nat. Med..

[141] Eisenberg T., Abdellatif M., Schroeder S., Primessnig U., Stekovic S., Pendl T., Harger A., Schipke J., Zimmermann A., Schmidt A. (2016). Cardioprotection and Lifespan Extension by the Natural Polyamine Spermidine. Nat. Med..

[142] Tan M., Yin Y., Ma X., Zhang J., Pan W., Tan M., Zhao Y., Yang T., Jiang T., Li H. (2023). Glutathione System Enhancement for Cardiac Protection: Pharmacological Options against Oxidative Stress and Ferroptosis. Cell Death Dis..

[143] Zhang H., Ryu D., Wu Y., Gariani K., Wang X., Luan P., D’Amico D., Ropelle E.R., Lutolf M.P., Aebersold R. (2016). NAD+ Repletion Improves Mitochondrial and Stem Cell Function and Enhances Life Span in Mice. Science.

[144] Palikaras K., Lionaki E., Tavernarakis N. (2015). Coordination of Mitophagy and Mitochondrial Biogenesis during Ageing in C. Elegans. Nature.

[145] Scarpulla R.C., Vega R.B., Kelly D.P. (2012). Transcriptional Integration of Mitochondrial Biogenesis. Trends Endocrinol. Metab..

[146] Colman R.J., Beasley T.M., Kemnitz J.W., Johnson S.C., Weindruch R., Anderson R.M. (2014). Caloric Restriction Reduces Age-Related and All-Cause Mortality in Rhesus Monkeys. Nat. Commun..

[147] Kemnitz J.W. (2011). Calorie Restriction and Aging in Nonhuman Primates. ILAR J..

[148] Lagouge M., Argmann C., Gerhart-Hines Z., Meziane H., Lerin C., Daussin F., Messadeq N., Milne J., Lambert P., Elliott P. (2006). Resveratrol Improves Mitochondrial Function and Protects against Metabolic Disease by Activating SIRT1 and PGC-1alpha. Cell.

[149] Fan W., Evans R.M. (2017). Exercise Mimetics: Impact on Health and Performance. Cell Metab..

[150] Chan D.C. (2020). Mitochondrial Dynamics and Its Involvement in Disease. Annu. Rev. Pathol..

[151] Ruiz A., Alberdi E., Matute C. (2018). Mitochondrial Division Inhibitor 1 (Mdivi-1) Protects Neurons against Excitotoxicity through the Modulation of Mitochondrial Function and Intracellular Ca^2+^ Signaling. Front. Mol. Neurosci..

[152] Poznyak A.V., Kirichenko T.V., Borisov E.E., Shakhpazyan N.K., Kartuesov A.G., Orekhov A.N. (2022). Mitochondrial Implications in Cardiovascular Aging and Diseases: The Specific Role of Mitochondrial Dynamics and Shifts. Int. J. Mol. Sci..

[153] Murphy M.P., Smith R.A.J. (2007). Targeting Antioxidants to Mitochondria by Conjugation to Lipophilic Cations. Annu. Rev. Pharmacol. Toxicol..

[154] Gioscia-Ryan R.A., Battson M.L., Cuevas L.M., Eng J.S., Murphy M.P., Seals D.R. (2018). Mitochondria-Targeted Antioxidant Therapy with MitoQ Ameliorates Aortic Stiffening in Old Mice. J. Appl. Physiol..

[155] Olgar Y., Billur D., Tuncay E., Turan B. (2020). MitoTEMPO Provides an Antiarrhythmic Effect in Aged-Rats through Attenuation of Mitochondrial Reactive Oxygen Species. Exp. Gerontol..

[156] Chiao Y.A., Zhang H., Sweetwyne M., Whitson J., Ting Y.S., Basisty N., Pino L.K., Quarles E., Nguyen N.-H., Campbell M.D. (2020). Late-Life Restoration of Mitochondrial Function Reverses Cardiac Dysfunction in Old Mice. eLife.

[157] Zikaki K., Kiachaki E., Gaitanaki C., Aggeli I.-K. (2025). “Villains” Turning Good: Antimycin A and Rotenone, Mitochondrial Respiratory Chain Inhibitors, Protect H9c2 Cardiac Cells Against Insults Triggering the Intrinsic Apoptotic Pathway. Int. J. Mol. Sci..

[158] Miwa S., Kashyap S., Chini E., von Zglinicki T. (2022). Mitochondrial Dysfunction in Cell Senescence and Aging. J. Clin. Investig..

[159] Symersky J., Osowski D., Walters D.E., Mueller D.M. (2012). Oligomycin Frames a Common Drug-Binding Site in the ATP Synthase. Proc. Natl. Acad. Sci. USA.

[160] Cadenas S. (2018). Mitochondrial Uncoupling, ROS Generation and Cardioprotection. Biochim. Biophys. Acta Bioenerg..

[161] Patyal P., Nguyen B., Zhang X., Azhar G., Ameer F.S., Verma A., Crane J., Kc G., Che Y., Wei J.Y. (2022). Rho/SRF Inhibitor Modulates Mitochondrial Functions. Int. J. Mol. Sci..

[162] Patyal P., Zhang X., Verma A., Azhar G., Wei J.Y. (2024). Inhibitors of Rho/MRTF/SRF Transcription Pathway Regulate Mitochondrial Function. Cells.

[163] Masuzawa A., Black K.M., Pacak C.A., Ericsson M., Barnett R.J., Drumm C., Seth P., Bloch D.B., Levitsky S., Cowan D.B. (2013). Transplantation of Autologously Derived Mitochondria Protects the Heart from Ischemia-Reperfusion Injury. Am. J. Physiol. Heart Circ. Physiol..

[164] Yin T., Luo J., Huang D., Li H. (2022). Current Progress of Mitochondrial Genome Editing by CRISPR. Front. Physiol..

[165] Song M., Ye L., Yan Y., Li X., Han X., Hu S., Yu M. (2024). Mitochondrial Diseases and mtDNA Editing. Genes Dis..

[166] Chen L., Zhou M., Li H., Liu D., Liao P., Zong Y., Zhang C., Zou W., Gao J. (2023). Mitochondrial Heterogeneity in Diseases. Signal Transduct. Target. Ther..

[167] Gropman A.L., Uittenbogaard M.N., Chiaramello A.E. (2024). Challenges and Opportunities to Bridge Translational to Clinical Research for Personalized Mitochondrial Medicine. Neurotherapeutics.

[168] Li Y., Li S., Qiu Y., Zhou M., Chen M., Hu Y., Hong S., Jiang L., Guo Y. (2022). Circulating FGF21 and GDF15 as Biomarkers for Screening, Diagnosis, and Severity Assessment of Primary Mitochondrial Disorders in Children. Front. Pediatr..

[169] Mengozzi A., Armenia S., De Biase N., Punta L.D., Cappelli F., Duranti E., Nannipieri V., Remollino R., Tricò D., Virdis A. (2025). Circulating Mitochondrial DNA Signature in Cardiometabolic Patients. Cardiovasc. Diabetol..

[170] Steele H.E., Horvath R., Lyon J.J., Chinnery P.F. (2017). Monitoring Clinical Progression with Mitochondrial Disease Biomarkers. Brain.

